# Preservation of satellite cell number and regenerative potential with age reveals locomotory muscle bias

**DOI:** 10.1186/s13395-021-00277-2

**Published:** 2021-09-04

**Authors:** Robert W. Arpke, Ahmed S. Shams, Brittany C. Collins, Alexie A. Larson, Nguyen Lu, Dawn A. Lowe, Michael Kyba

**Affiliations:** 1 Lillehei Heart Institute and Department of Pediatrics, Medical School, University of Minnesota, Cancer and Cardiovascular Research Building, 2231 6th Street SE, Minneapolis, MN 55455 USA; 2 Present address: Division of Biological Sciences, University of Missouri, 105 Tucker Hall, Columbia, MO 65211 USA; 3 Human Anatomy and Embryology Department, Faculty of Medicine, Suez Canal University, Ismailia, Egypt; 4 Divisions of Rehabilitation Science and Physical Therapy, Department of Rehabilitation Medicine, Medical School, University of Minnesota, Minneapolis, MN 55455 USA; 5 Present address: Department of Human Genetics, University of Utah, 15 North 2030 East, Salt Lake City, UT 84112 USA; 6 Department of Integrative Biology and Physiology, Medical School, University of Minnesota, Minneapolis, MN USA

**Keywords:** Satellite cells, Regeneration, Transplantation

## Abstract

**Background:**

Although muscle regenerative capacity declines with age, the extent to which this is due to satellite cell-intrinsic changes vs. environmental changes has been controversial. The majority of aging studies have investigated hindlimb locomotory muscles, principally the tibialis anterior, in caged sedentary mice, where those muscles are abnormally under-exercised.

**Methods:**

We analyze satellite cell numbers in 8 muscle groups representing locomotory and non-locomotory muscles in young and 2-year-old mice and perform transplantation assays of low numbers of hind limb satellite cells from young and old mice.

**Results:**

We find that satellite cell density does not decline significantly by 2 years of age in most muscles, and one muscle, the masseter, shows a modest but statistically significant increase in satellite cell density with age. The tibialis anterior and extensor digitorum longus were clear exceptions, showing significant declines. We quantify self-renewal using a transplantation assay. Dose dilution revealed significant non-linearity in self-renewal above a very low threshold, suggestive of competition between satellite cells for space within the pool. Assaying within the linear range, i.e., transplanting fewer than 1000 cells, revealed no evidence of decline in cell-autonomous self-renewal or regenerative potential of 2-year-old murine satellite cells.

**Conclusion:**

These data demonstrate the value of comparative muscle analysis as opposed to overreliance on locomotory muscles, which are not used physiologically in aging sedentary mice, and suggest that self-renewal impairment with age is precipitously acquired at the geriatric stage, rather than being gradual over time, as previously thought.

**Supplementary Information:**

The online version contains supplementary material available at 10.1186/s13395-021-00277-2.

## Background

Skeletal muscle is a highly regenerative tissue. The lifelong capacity for regeneration depends on a population of satellite cells, located peripheral to the muscle fiber but under its basal lamina [1]. Satellite cells proliferate in response to injury to produce a pool of myoblasts that will fuse to form new muscle fibers as well as replacement cells to maintain the stem cell pool [2–4], a process referred to as self-renewal [5]. Although the regenerative properties of satellite cells are under intensive investigation, essential questions about the regulation of the satellite cell compartment remain, for example what specifies the number of satellite cells per muscle.

Both skeletal muscle mass and the potential of muscle to regenerate after injury decline with age; however, the role of changes intrinsic to the satellite cells in these declines has been controversial. Numerous groups have documented age-related decreases in satellite cell numbers in mice [6–12]. Some groups have documented no significant reduction [13] or no change [14]. Pax7 is selectively expressed by satellite cells and required for their maintenance [15]; therefore in these studies, satellite cell numbers, generally from locomotory muscles, were quantified by counting Pax7+ cells in representative muscle sections or representative isolated single myofibers and extrapolating based on estimated number of sections or fibers per muscle. We considered that perhaps some of this discordance is from sampling bias inherent in methods that quantify by extrapolating from a subset, as well as the potential for systematic errors of extrapolation. For this reason, in the current study, we investigate satellite cell numbers across a variety of locomotory and non-locomotory muscles in young *vs.* old mice using the holistic approach of counting all of the Pax7+ cells present in particular muscles by flow cytometry using the Pax7-ZsGreen BAC transgenic strain, in which satellite cells are labeled green fluorescent [16].

The literature also contains significant discordance on whether the effects of aging are mainly mediated through alterations in the satellite cells or in their environment. There are reports demonstrating the intrinsic regenerative potential of the satellite cell pool being impaired with age [12, 17–19]. Other studies argue that although the number of satellite cells is decreased in aged muscle, the intrinsic myogenic potential and self-renewal capacity of satellite cells remains unaltered [11], that aged donor satellite cells are as functional as those from young donors [8, 20–22], and that environmental factors rather than cell intrinsic changes are responsible for the impaired regeneration in aged animals [7, 14, 23–28].

In order to measure both the intrinsic self-renewal and differentiation potential of a population of satellite cells, we have employed a two armed transplantation assay in which a defined number of satellite cells is simultaneously transplanted into both tibialis anterior (TA) muscles of immunodeficient, dystrophin-deficient NSG-mdx^4Cv^ [29] recipient mice. One limb is used for FACS to count the number of undifferentiated (ZsGreen+) satellite cells 1 month post-transplant, giving a quantitative value to self-renewal, or the ability to contribute to the satellite cell pool, while the other is processed for histology to count dystrophin+ fibers, giving a quantitative value to the ability of the donor cells to generate fibers (differentiation potential). We apply these assays to evaluate self-renewal and differentiation potential in the context of aging.

## Methods

### Mice

Satellite cells were isolated from Pax7-ZsGreen male mice [16] crossed >15 generations to a C57BL/6 background or C57BL/6 mice. Transplant recipients were *NSG-mdx*^*4Cv*^ mice [29]. Wild-type C57BL/6 mice were obtained from the aging rodent colony of the National Institute on Aging. All procedures were carried out in accordance with protocols approved by the University of Minnesota Institutional Animal Care and Use Committee.

### Mouse satellite cell harvest

Bulk isolation of satellite cells was performed as described previously [29, 30]. Muscle was carefully dissected. With a razor blade parallel to the muscle fibers, forceps were used to separate the fibers. The muscle was incubated shaking for 75 min in 0.2% collagenase type II (Gibco, Grand Island, NY) in high glucose Dulbecco’s modified Eagle’s medium (DMEM) containing 4.00 mM L-glutamine 4500 mg/L glucose, and sodium pyruvate (HyClone, Logan, UT) supplemented with 1% Pen/Strep (Gibco) at 37 °C. Samples were washed two times with Rinsing Solution (F-10+), Ham’s/F-10 medium (HyClone) supplemented with 10% Horse Serum (HyClone), 1% 1 M HEPES buffer solution (Gibco), and 1% Pen/Strep, and pulled into a sheared Pasteur pipette. The samples were centrifuged and washed again. After aspiration, the sample was resuspended in F-10+ containing collagenase type II and dispase (Gibco), vortexed, and incubated shaking at 37 °C for 30 min. Samples were vortexed again, drawn and released into a 10 mL syringe with a 16-gauge needle four times, then with a 18-gauge needle four times to release the cells from the muscle fibers prior to passing the cell suspension through a 40-μm cell strainer (Falcon, Hanover Park, IL). The sample was drawn and released into a 10-mL syringe with the 18-gauge needle four additional times and passed through a new 40-μm cell strainer. After centrifuging, the samples were resuspended in Fluorescent-activated Cell Sorting (FACS) staining medium: Phosphate Buffered Saline (PBS, Corning, Manassas, VA) containing 2% fetal bovine serum (HyClone) and 0.5 μg/mL propidium iodide, for FACS analysis and sorting on a FACSAriaII (BD Biosciences, San Diego, CA).

Quantification of satellite cells from single muscle groups was performed similarly. Digested muscle samples were drawn and expelled into a 3-mL syringe four times through a 16-gauge, then four times through an 18-gauge needle. The cell suspension was passed through a 40-μm cell strainer. Three milliliters of F10+ was added to each sample to prevent over-digestion, and samples were centrifuged, then resuspended in FACS staining medium. For transplanted TAs, the samples were stained using an antibody mixture of PE-Cy7 rat anti-mouse CD31 (clone 390), PE-Cy7 rat anti-mouse CD45 (clone 30-F11), Biotin rat anti-mouse CD106 (clone 429(MVCAM.A)), and PE Streptavidin from BD Biosciences (San Diego, CA) and Itga7 647 (clone R2F2) from AbLab (Vancouver, B.C., Canada). The number of donor (ZsGreen +) satellite cells and total satellite cells (lineage negative; VCAM, Itga7 double positive cells) was determined by running the entire volume through the FACS and recording all events.

### Antibodies for FACS analysis

For additional characterization of satellite cells, we stained for lineage negative; CXCR4 and Itgb1 double positive cells. PE-Cy7 anti-mouse TER-119 (clone TER-119), APC anti-mouse/rat CD29 (clone eBioHMb1-1), and PE-Cy7 anti-mouse Ly6A/E (Sca-1) (clone D7) from eBioscience (San Diego, CA); PE-Cy7 rat anti-mouse CD11b (clone M1/70), PE-Cy7 CD45, and Biotin rat anti-mouse CD184 from BD Biosciences antibodies were incubated with samples on ice for 1 h. Additionally, we stained for lineage negative; CD34, Itga7 double-positive cells. PE-Cy7 Rat anti-mouse CD11b (clone M1/70), PE-Cy7 anti-mouse Ly6A/E (Sca-1) (clone D7), and Biotin anti-mouse CD34 (clone RAM34) from eBioscience (San Diego, CA) and Itga7 PE (clone R2F2) from AbLab (Vancouver, B.C., Canada) antibodies were incubated for 1 h on ice. After this incubation, samples were washed and resuspended with FACS staining medium. PE or APC conjugated Streptavidin reagents, respectively, were incubated on ice for 20 min. Samples were washed and resuspended with FACS staining medium for FACS analysis.

### Flow cytometric counting

For quantification of satellite cells, cells from individual muscles were resuspended in 200 μL FACS staining medium, with the exception of gastrocnemius and diaphragm which were resuspended in 400 μL in order to keep total cell concentrations similar. Samples were run out completely on a BD FACS Aria II, with red (641 nm), blue (488 nm), and yellow-green (561 nm) lasers. Propidium iodide-negative (live) cells were gated into either SSC *vs.* ZsGreen for unstained samples, or for stained samples: APC (Itga7) *vs.* PE-Cy7 (Lin), gating Lin-neg cells into APC (Itga7) *vs.* PE (VCAM), gating double-positive cells into SSC *vs.* ZsGreen, and counting ZsGreen+ cells, as shown in Fig. 2b.

### Pax7 immunostaining

Slides were fixed using 4% paraformaldehyde (PFA) at room temperature for 5 min, air dried, and rehydrated using PBS for 5 min followed by antigen retrieval in citrate Buffer (1.8 mM Citric Acid and 8.2 mM Sodium Citrate in water) using a pressure cooker. Slides were boiled in a Coplin jar for 30 min, then rinsed with cold tap water for 10 min, washed twice with PBS, 5 min each. After antigen retrieval, sections were circled with nail polish, dried, then incubated with 3% H_2_O_2_ in PBS for 5 min, and washed twice with PBS, 5 min each. Sections were blocked using 0.5% PerkinElmer Blocking Reagent (Cat # FP1020) in PBS for 1 h at RT followed by overnight incubation at 4 °C with primary antibodies, mouse anti-mouse Pax7 from the Developmental Studies Hybridoma Bank, and polyclonal rabbit anti-mouse laminin (Sigma-Aldrich L9393) in blocking buffer. Secondary cocktail, goat anti-mouse IgG1 Biotin (Jackson Immunoresearch Cat# 115-065-205), and goat anti-rabbit IgG H&L Alexa Fluor 488 (Sigma-Aldrich. Cat # A11034) in blocking buffer were applied for 2 h at RT. Following 2 PBS washes, slides were incubated with Vectastain ABC reagent (Vectorlab Cat # PK-6100) for 3 h followed by PBS wash. Finally, slides were incubated 10 min in the dark with Tyramide signal amplification (TSA Cyanine 3 Cat # NEL744) in blocking buffer, washed with PBS, and mounted with anti-fade Prolong gold with DAPI.

### Muscle injury and transplantation

As described previously [29], 48 h prior to intramuscular transplantation of cells, approximately 4-month-old *NSG-mdx*^*4Cv*^ mice were anesthetized with ketamine and xylazine and both hind limbs were subjected to a 1200 cGy dose of irradiation using an RS 2000 Biological Research Irradiator (Rad Source Technologies, Inc., Suwanee, GA). Lead shields limited exposure to the hind limbs only. Twenty-four hours prior to transplant, 15 μL of cardiotoxin (10 μM in PBS, Sigma, Saint Louis, MO) was injected into the right and left TAs of each mouse with a Hamilton syringe to induce injury. Twenty-four hours later, a defined number of satellite cells was collected by FACS and resuspended in a volume such that the indicated number of cells per injection was present in 10 μL of sterile saline. Each mouse was then injected with 10 μL into each TA. Four weeks after transplantation, one transplanted TA of each mouse was harvested and prepared for sectioning and staining as described previously [29], while the other transplanted TA was prepared for FACS analysis as described above.

### Colony-forming assays

Satellite cells were identified by FACS and single cells were sorted into gelatin-coated wells of a 96-well plate containing 150 μL of mouse myoblast medium: DMEM/F-12 medium without L-glutamine (Cell Gro, Manassas, VA) containing 20% Fetal Bovine Serum (HyClone), 10% Horse Serum (HyClone), 10 ng/mL human basic fibroblast growth factor (R&D Systems, Minneapolis, MN), 1% Pen/Strep, 1% Glutamax (Gibco), and 0.5% Chick Embryonic Extract (US Biological, Swampscott, MA).

Plates were cultured for 8 days under physiological oxygen growth conditions (5% O_2_, 5% CO_2_, 90% N_2_) at 37 °C in a tissue culture incubator. Wells containing colonies were identified and fixed with 4% paraformaldehyde for 20 min at room temperature. For immunostaining, cells were permeablized with 0.3% triton-X100 for 20 min at room temperature, washed once with PBS, and blocked with 3% bovine serum albumin (BSA) in PBS for 1 h at room temperature. Colonies were stained with a 1:20 dilution of MF 20 antibody supernatant (recognizing sarcomeric myosin, obtained from the Developmental Studies Hybridoma Bank, University of Iowa) in 3% BSA in PBS overnight at 4 °C. Plates were washed three times with PBS and incubated with a 1:500 dilution of Alexa Fluor 555 goat anti-mouse secondary antibody (Life Technologies) for 45 min at room temperature. The plates were washed four additional times with PBS before the addition of a 1:1000 dilution of 4,6-diamidino-2-phenylindole (DAPI) in PBS for 20 min at room temperature. After one final wash with PBS, the cells were covered with PBS. 10× magnification images were taken on a Zeiss Observer.Z1 inverted microscope equipped with an AxioCam MRm camera (Thornwood, NY). Merged images were analyzed with the open-source algorithm, G-Tool [31]. This software was utilized to determine the number of nuclei in a satellite cell colony and the percentage of nuclei in myosin heavy chain (MHC) positive cytoplasm.

### Histology and dystrophin/laminin immunofluorescence

The TA was removed and placed in OCT Compound (Scigen Scientific, Gardena, CA), frozen in liquid nitrogen-cooled 2-methylbutane (Sigma), and stored at −80 °C. Ten-micrometer cryosections were cut on a Leica CM3050 S cryostat (Leica Microsystems, Buffalo Grove, IL). Cryosections were fixed with ice cold acetone, air dried, rehydrated with PBS, and blocked for 1 h with 3% BSA in PBS. After incubating slides for 1 h at RT with a rabbit polyclonal antibody to dystrophin (Abcam, Cambridge, MA) and a mouse monoclonal antibody to laminin (Sigma – clone LAM-89), the sections were incubated for 45 min at RT with Alexa Fluor 555 goat anti-rabbit and Alexa Fluor 488 goat anti-mouse IgG antibodies (Life Technologies, Grand Island, NY). Coverslips were mounted with Immu-Mount (Thermo Scientific, Kalamazoo, MI). Slides were imaged with a Zeiss Axio Imager.M2 with an AxioCam MRm camera (Carl Zeiss Microscopy, LLC, Thornwood, NY). The numbers of donor (dystrophin+) and total (laminin+) muscle fibers were determined after merging tiled images of the entire cross-section from transplanted TAs using Photoshop and manually counting the fibers.

### In vitro assessment of TA muscle

As described previously [32, 33], mice were anesthetized with an intraperitoneal injection of Avertin (250 mg/kg). The distal tendon of the TA muscle and the knee were attached by silk suture to a force transducer. TA muscles were then transferred into an organ bath containing mammalian Ringer solution: 120.5 mM NaCl; 20.4 mM NaHCO_3_; 10 mM glucose; 4.8 mM KCl; 1.6 mM CaCl_2_; 1.2 mM MgSO_4_; 1.2 mM NaH2PO_4_; 1.0 mM pyruvate, adjusted to pH 7.4 perfused continuously with 95% O_2_ / 5% CO_2_. The isolated TA muscles were stimulated by an electric field generated between two platinum electrodes placed longitudinally on either side of the TA muscles (using square wave pulses at 25 V amplitude, 0.2 ms in duration, 150 Hz). Muscles were maintained at the optimum length (*Lo*) during the determination of isometric twitch force with a 5-min recovery period between stimulations. Optimal muscle length (*Lo*) and stimulation voltages (25 V) were chosen based on muscle length manipulation and a series of twitch contractions that generated the maximum isometric twitch force. After adjusting the optimal muscle length (*Lo*) and measuring the maximum isometric tetanic force, total muscle cross-sectional area (CSA) was calculated by dividing muscle mass (mg) by the product of muscle length (mm) and 1.06 mg/mm^3^, the mammalian skeletal muscle density. Specific force (*sF*o) was then calculated by normalizing maximum isometric tetanic force to CSA.

### Statistics

Data were analyzed by two-tailed *t*-tests and reported as means with standard errors. Differences were considered significant at the *α*<0.05 level.

## Results

### Changes in satellite cell number over eight muscles with age

**Fig. 1:**
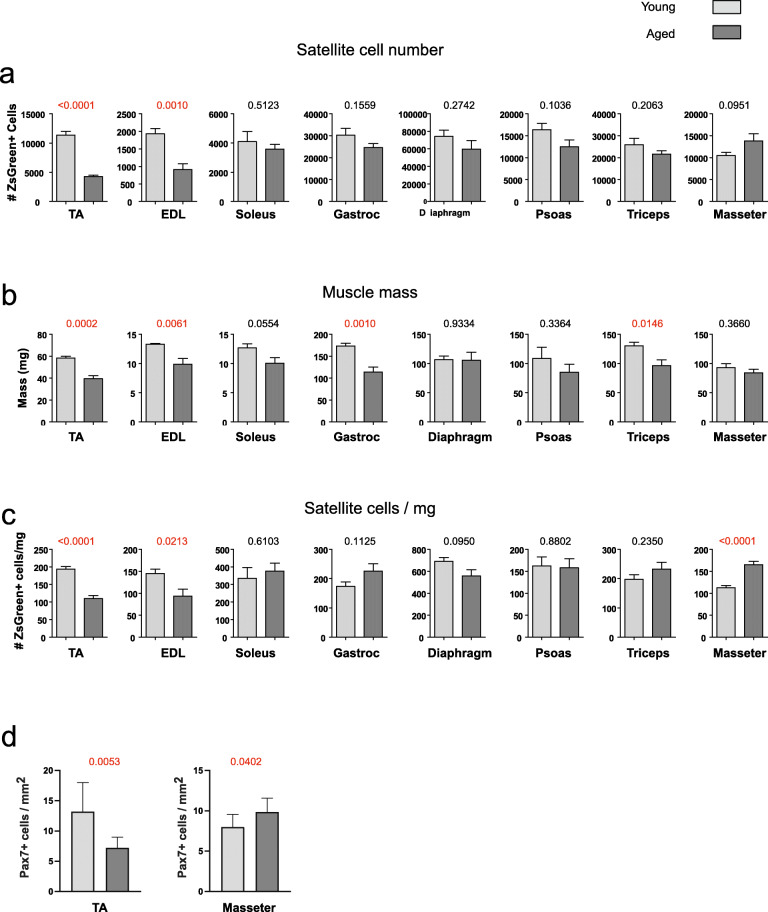
Comparisons of eight different muscle groups from 4-month- and 2-year-old male Pax7-ZsGreen mice. **a** Quantification of the number of satellite cells in eight different muscle groups by FACS. **b** Measured muscle mass in milligrams. **c** Number of satellite cells normalized to muscle mass. Data shown are mean ± SE (*n*=6, except diaphragm where *n*=4). Results of *p*-value comparisons performed using two-tailed t tests are indicated above each graph

In order to obtain a comprehensive view of the size of the satellite cell compartment and changes with age, we investigated eight muscle groups representing different activities: locomotory, masticatory, and respiratory in young (4 months) and old (2 years) male C57BL/6 mice (Fig. 1). Importantly, each individual muscle was processed in such a way that that there was no residual tissue after processing, i.e., every cell and fiber fragment was suspended, and the sample drained, so that the entire muscle digest passed through the flow cytometer. Representative FACS profiles used for the quantification of the total number of satellite cells in the different muscle groups from 4-month- and 2-year-old Pax7-ZsGreen mice are shown in Additional file 1. In most muscles, both satellite cell number (Fig. 1a) and muscle mass (Fig. 1b) declined modestly at 2 years of age, and in concert. The masseter, surprisingly, trended toward an increase in total satellite cell number (>25% increase, *p* < 0.0951, Fig. 1a) in aged mice. Only two muscles, the TA and EDL (extensor digitorum longus), showed large declines in satellite cell number that were statistically significant, while the remaining limb muscles and diaphragm showed modest declines of between 5 and 20% that were not statistically significant. We investigated age-related loss of muscle mass, sarcopenia, and found that only limb muscles displayed reduced average mass, with TA, EDL, gastrocnemius, and triceps significantly reduced (by about 35%) and soleus and psoas trending lower with age (Fig. 1b). It is notable that the most excessive changes occur in the locomotory muscles, and given that caged mice are sedentary, their aging in the laboratory might not reflect the course of aging under normal conditions. When satellite cell number was normalized to muscle mass to obtain a measure of satellite cell density, only the TA and EDL showed significant declines while the diaphragm trended lower in the 2-year-old samples (*p*=0.1) (Fig. 1c). No change in satellite cell density was observed in the soleus and psoas samples; density trended higher with age in the gastrocnemius and triceps, and remarkably, satellite cell density was significantly increased (*p*<0.0001) in the masseter (Fig. 1c).

To validate the unexpected finding that masseter showed an increased number of satellite cells in aged mice, we used an independent assay (immunohistochemical staining for Pax7 and laminin) on the TA and masseter muscles of an independent cohort of 3-month and 22-month-old wild-type mice from another institution (Additional file 2). Immunostaining for Pax7 in this cohort revealed again an increase in satellite cell density with age in the masseter, in contrast to the decline in satellite cell density seen in the TA muscle (Fig. 1d).

### Ex vivo clonal assays of aged vs. young cells

We began investigating cell-autonomous functional differences by evaluating the colony-forming potential of single cells from each muscle group at both ages. Cloning efficiency, an integrated measurement of the ability of cells to survive, proliferate, and form colonies in vitro, decreased significantly in almost every muscle between 4 months and 2 years of age, including strong trends in the soleus, diaphragm, and masseter muscle (Additional file 3 a). Differences in colony size were more notable between muscles than between ages, with diaphragm satellite cells giving the largest colonies, as was observed previously [31]. A significant decline in colony size with age was observed in the gastrocnemius, diaphragm, psoas, and triceps, while a moderate increase was seen in the TA (Additional file 3 b). The rate of differentiation (% of nuclei in MHC+ cytoplasm) was unchanged in most muscles, but was significantly reduced with age in the TA, EDL, and psoas muscles (Additional file 3c).

### Assaying self-renewal in vivo

**Fig. 2:**
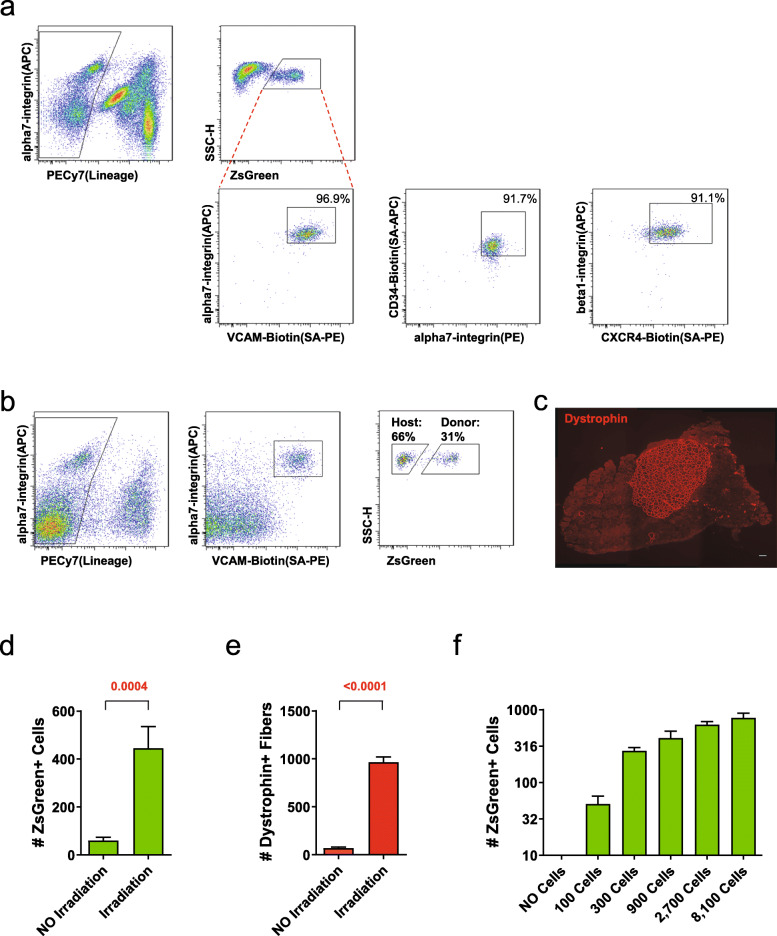
Assaying satellite cell self-renewal and differentiation in vivo. **a** Representative FACS profile showing the percentage of ZsGreen+ cells that are lineage negative, double positive cells for the three most-commonly used antibodies to identify satellite cells (*n*=3). **b** Representative FACS profile for the isolation of purified satellite cells from transplanted muscle. Lineage-negative gating (against CD31 and CD45, both PE-Cy7 conjugated) is shown at left. Positive gating for VCAM-1 (PE), Itga7 (APC) is shown in the middle, and ZsGreen within the Lin– VCAM+Itga7+ population is shown at right. **c** Representative image showing Dystrophin (red) expression 4 weeks after transplant of 300 Pax7-ZsGreen cells. Scale bar is 100 μm. **d** FACS quantification of the number of ZsGreen+ cells after transplant with or without irradiation. Data shown are mean ± SE (*n*=12 transplanted TAs/group). Statistical comparisons were performed using two-tailed t tests. Note that irradiation significantly enhances contribution to the satellite cell pool. **e** Quantification of the total number of Dystrophin+ fibers 4 weeks after transplant in the presence/absence of irradiation. Shown are mean ± SE (*n*=12 transplanted TAs/group). Statistical comparisons were performed using two-tailed t tests. **f** Quantification of the number of ZsGreen+ cells 4 weeks after transplant of different numbers of ZsGreen+ cells. Shown are mean ± SE (*n*=6 transplanted TAs)

To develop a quantitative readout of self-renewal based on the ability of transplanted satellite cells to contribute to the stem cell compartment of engrafted muscle, we first evaluated age-dependence of surface markers that have been described to define this compartment. Satellite cells from Pax7-ZsGreen/BL6 mice were evaluated for either alpha7-integrin (Itga7) and VCAM [34, 35]; Itga7 and CD34 [4]; or beta1-integrin (Itgb1) and CXCR4 [36] in conjunction with lineage-negative staining for hematopoietic and endothelial markers. We observed that 97% of the ZsGreen+ satellite cells were Itga7/VCAM double positive and >91% were positive for the other Itga7/CD34 or Itgb1/CXCR4 (Fig. 2a). The inverse analysis gave similar results with >90% of double positive (DP) cells being ZsGreen+ (Additional file 4). We next examined whether the surface marker profile of satellite cells changed in aged mice. ZsGreen+ satellite cells isolated from 2-year-old+ Pax7-ZsGreen mice (Additional file 5) showed a similar surface marker profile to that of 4-month-old mice. For the studies below involving transplantation of ZsGreen+ cells, we use Itga7/VCAM to identify the total (host + donor) pool.

To monitor both self-renewal and differentiation potential of transplanted cell populations, we irradiated and injured both TA muscles of immunodeficient, dystrophin-deficient NSG-mdx^4Cv^ mice and transplanted each with 300 Pax7-ZsGreen cells. The 15 μl cardiotoxin injection causes the destruction and regeneration of approximately 40% of the entire TA muscle (Additional file 6), providing ample space for injected satellite cells to contribute to both regeneration and self-renewal. One month after transplantation, both TA muscles were dissected; one was processed for FACS and the other for histology. FACS analysis demonstrated that a donor ZsGreen+ cell subpopulation was present within the Lin– VCAM+Itga7+ satellite cell pool; thus some portion of the progeny of the donor cells contributed to renewal of the satellite cell pool (Fig. 2b). Histology demonstrated many dystrophin+ fibers in the other TA, revealing the differentiation potential of the transplanted cells (Fig. 2c).

Skeletal muscle transplantation experiments frequently incorporate irradiation to blunt the response of the host satellite cells, resulting in greater contribution of both fibers and associated satellite cells [20, 21]. Irradiation also leads to extensive proliferation of donor stem cells and their progeny (myoblasts) [37]. Recent work suggests that activation of the innate immune system within irradiated muscle plays a role in enhancing donor satellite cell engraftment [38]. To measure the necessity of irradiation in our system, we performed transplantations of 300 cells with and without irradiation and found that both contribution to the satellite cell pool and to new fibers were significantly reduced without irradiation (8-fold and 16-fold respectively, Fig. 2d, e).

The effect of irradiation hints at the possibility of competition between satellite cells for contribution to the post-regeneration satellite cell pool, although this has never formally been demonstrated. An accurate assay for the intrinsic self-renewal potential of a cell population requires that contribution to the satellite cell pool not be confounded with the effects of competition between donor cells. To determine whether and how this assay could be used to read out intrinsic self-renewal potential, we performed a dose-response transplantation experiment, starting with 100 donor cells, going up in 3-fold steps to 8100 cells (Fig. 2f). This revealed a severe non-linearity in contribution at cell numbers 900 or greater, i.e., the increase in contribution of 300 cells over 100 cells is close to 300%, while the increase in contribution of 900 cells over 300 cells is only about 50%, and even less at 2700 and so on. This indicates two important points: first, this assay needs to be performed at numbers close to 300 cells or fewer in order for changes in the contribution rate to correlate linearly with changes in the rate of self-renewal. At 900 cells and higher, significant changes in self-renewal read out as minor changes in ZsGreen+ cell numbers. Second, the donor cells are competing with each other for contribution to the most accessible space. To avoid internal competition, the number of transplanted cells needs to be smaller than the space available, hence our use of 300 cells per TA muscle.

### Aging to 2 years does not significantly impair in vivo self-renewal potential of C57BL/6 satellite cells

**Fig. 3:**
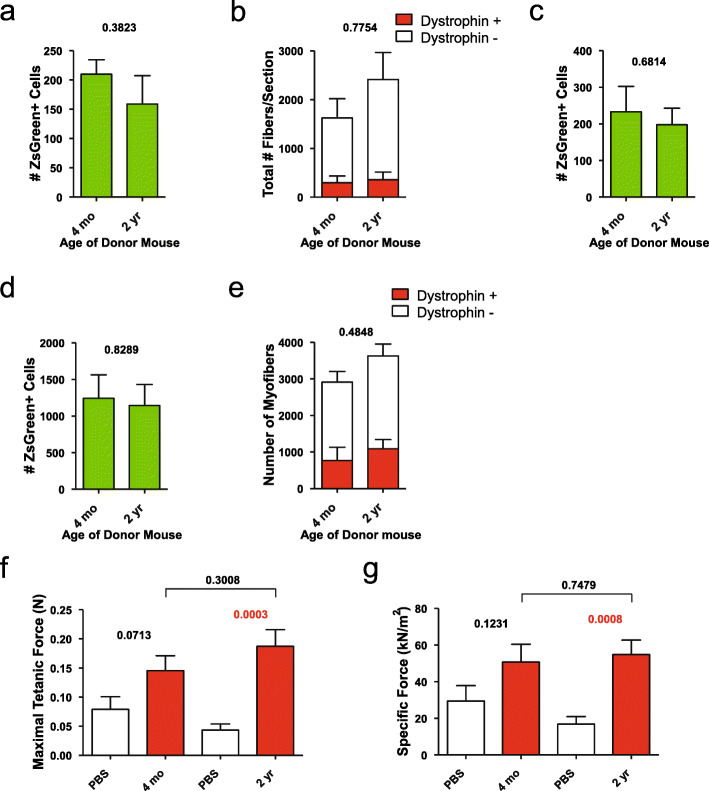
Transplant of ZsGreen+ cells from 4-month- or 2-year-old Pax7-ZsGreen mice into irradiated and cardiotoxin-injured *NSG-mdx*^*4Cv*^ mice. **a** Quantification by FACS of the number of ZsGreen+ cells 4 weeks after transplant of 100 ZsGreen+ cells (*n*=4). **b** Quantification of the number of Dystrophin+ fibers (*n*=4), 4 weeks after transplant of 100 ZsGreen+ cells. **c** Quantification of the number of ZsGreen+ cells (*n*=6) after transplant of 300 ZsGreen+ cells. Data shown are mean ± SE. Statistical comparisons employed two-tailed t tests. **d** Quantification of the number of ZsGreen+ cells 4 weeks after transplant of 2700 ZsGreen+ cells (*n*=4). **e** The number of Dystrophin+ fibers 4 weeks after transplant of 2700 ZsGreen+ cells (*n*=4). **f** Maximal tetanic force 4 weeks after transplant of 2700 ZsGreen+ cells (*n*=8). **g** Specific force measurements 4 weeks after transplant of 2700 ZsGreen+ cells (*n*=8)

We next applied this assay to measure the self-renewal potential of satellite cells from aged (2-year-old) *vs.* young (4-month-old) C57BL/6 mice. We first tested two doses, both in the linear range: 100 and 300 cells into irradiated, cardiotoxin-injured NSG-mdx4Cv mice (Fig. 3a–c). Neither dose demonstrated a statistically significant reduction in self-renewal potential, nor in fiber differentiation potential. These low numbers of donor cells do not produce a detectable increase in contractile function of transplanted muscles [29]; therefore, we also performed transplantations of 2700 cells. Similar self-renewal and fiber differentiation potentials were seen at this cell number (Fig. 3d, e), and we found no difference in maximal tetanic or specific force in TA muscle regenerated with old cells *vs.* TA muscle regenerated with young cells (Fig. 3f, g).

We considered that because our mice are sedentary during their lifespans, their limb muscles might not be representative of muscles undergoing normal aging. Therefore, we decided to transplant satellite cells from a muscle under constant use—the diaphragm. Nine hundred ZsGreen+ satellite cells isolated from the diaphragm of young or old mice were transplanted into preconditioned TAs which were harvested either 6 weeks (Additional file 7 a, b) or 15 weeks (Additional file 7 c, d) after transplant. No significant differences in self-renewal or fiber contribution were observed at either time point, mirroring the results with hind limb muscle satellite cells, above.

## Discussion

In the present study, we use flow cytometry to quantify the number of undifferentiated Pax7-ZsGreen+ cells in individual muscles, both in the context of unmanipulated muscles of aged *vs.* young mice, as well as in the context of transplantation, where the Pax7-ZsGreen reporter serves both as an indicator of donor origin as well as of undifferentiated status. The FACS-based approach is very efficient; thus, we were able to quantify the satellite cell content of eight different muscles from each animal studied in the aging cohorts. As with any method, the FACS-based approach has caveats, principally that extraction needs to be efficient, and that it may be affected by the extent of ECM or fibrosis. Since aged muscle has greater ECM content, inefficient extraction might lead to underestimation of cell number in aged specimens.

We found that few muscle groups showed a statistically significant decline in mean numbers of satellite cells at 2 years of age. The exceptions were the TA and EDL, which experienced large (>50%), statistically significant declines at 2 years of age. Using other methodologies, previous studies have quantified the number of Pax7+ cells in freshly isolated myofibers from EDL [8, 9, 11, 22, 39], soleus [39], and TA [2]. Consistent with our results, these studies have found lower numbers of satellite cells in TA and EDL muscles of aged mice, and this has led to the generally-accepted notion in the field that satellite cell numbers decline significantly with age. However, considering a broader sampling of muscles, it is now clear that TA and EDL are not typical. This is unfortunate, because these two muscles are probably the most-studied muscles in the mouse system. Furthermore, age-related loss of muscle mass was much more extreme in the locomotory muscles, which is disconcerting because caged mice are abnormally sedentary. It raises the possibility that satellite cell compartment size changes over time in locomotory muscles of caged mice may be more extreme due to lack of normal use over time than to aging, per se. An increase in satellite cell content has been reported after endurance training in old rats [40] and mice [41]. After 8 weeks of progressive endurance training, the TAs of an old exercised group of mice had significantly more satellite cells than an old sedentary group [42]. At the very least, this implicates exercise as relevant to the loss of satellite cells seen in locomotory muscles in caged rodents. The diaphragm and masseter, muscles used more physiologically under laboratory conditions, did not lose mass with age and showed only very modest decline, or indeed in the case of masseter, an increase, in satellite cell numbers. Thus, our studies are not consistent with the notion of a gradual and progressive satellite cell number decline with age, at least not in the male C57BL/6 mouse model aged for 2years.

In older humans, a greater loss of muscle mass is observed in the lower limbs possibly due to a detraining effect because of the significant reduction in physical activity [43]. The age-related loss of muscle mass was much more extreme in the locomotory muscles. Looking specifically at human satellite cells, their activation in response to damaging and non-damaging exercise as well as their reduction in the context of disuse has been noted (see [44] for review). Aging in humans leads to preferential atrophy of type II muscle fibers, and type II fiber-associated satellite cells have been shown to decline with age [44]. A recent longitudinal study in humans found a decline in satellite cell density in females at the time of menopause, supporting the notion that an aging-related environmental change in older humans could be responsible for some degree of satellite cell decline [26]. Although not as severely constrained as caged mice, humans are also fairly sedentary, and it will be interesting to see whether the notion of relatively stable homeostatic maintenance of satellite cells within normally used muscles applies in the human context.

Comparing the ability of young and old satellite cells to contribute to the satellite cell pool after transplantation into injured muscle, i.e., measuring self-renewal potential, we found no difference between hind limb satellite cells from 4-month-old and 2-year-old mice. Although this result is consistent with original satellite cell aging literature [8, 11, 14, 23], it was nonetheless somewhat surprising, as several studies have suggested that intrinsic self-renewal potential declines with age [12, 17–19]. However, there are important distinctions to point out regarding the series of studies mentioned. The first is that one of the studies [12] found significant declines in “geriatric” mice of 2.5 years, but found by most parameters, including regeneration post-transplantation, that satellite cells from “old” mice (2 years, the typical aged cohort) were not significantly different from cells from young mice. This study showed that somewhere between 2 and 2.5 years, many satellite cells become senescent, which is quite distinct from a model in which cell intrinsic self-renewal potential declines gradually in non-senescent cells as they age. In showing little difference by 2 years, this study is actually consistent with our results and is not inconsistent with the interpretations of the earlier literature. Of the others, one of the studies derives results from transplants of 10,000 cells [19], and our dose-response data clearly shows that this is far outside of the linear range; thus, the differences may have had more to do with competition for space than with intrinsic self-renewal. To address the question of how much space is available for engraftment, we evaluated regeneration in unirradiated TA muscles injected with 15 μl of cardiotoxin and found that this space is at least 40% of the TA. Transplantation of myofiber-associated satellite cells, coupled with an induced muscle injury has been shown to engraft the entire TA [45]. Further implicating competition, in the study of Bernet and colleagues [17], recipient mice were not irradiated; thus, different rates of engraftment into the satellite cell compartment may relate to intrinsic differences in competitive ability rather than self-renewal per se. It is also reasonable to consider that there may be an environmental component to the rate of aging, whereby mice from some laboratories have satellite cells that enter the geriatric, senescence-prone phase slightly earlier than mice from other laboratories. Nevertheless, the data presented in the current study is consistent with the notion that up until the most geriatric stage, satellite cells show little age-related loss in their intrinsic ability to self-renew or regenerate.

## Conclusions

This work provides a comprehensive comparison of eight different muscle groups and shows that except for abnormally under-utilized locomotory muscles, aging to 2 years on the C57BL/6 background does not involve a gradual decline in satellite cell density. Upon transplantation into tibialis anterior muscles of immunodeficient dystrophic mice, transplanted cells contribute to both the satellite cell compartment and to newly generated dystrophin+ myofibers. There is negligible decline with age in the ability of transplanted cells to generate new satellite cells or new muscle fibers.

## Supplementary Information

**Additional file 1.** FACS plots for the quantification of the total number of satellite cells in different muscle groups from four-month- and two-year-old Pax7-ZsGreen mice. Representative FACS profiles for the gating for ZsGreen+ cells from eight different muscle groups – TA, EDL, Soleus, Gastrocnemius, Diaphragm, Psoas, Triceps and Masseter (n=6, except diaphragm where n=4).

**Additional file 2.** Representative immunostaining of Pax7+ cells, together with laminin and DAPI, in young (3 month) vs. old (22 months) TA muscle sections (above), and young vs. old masseter muscle sections (below). Scale bar: 50 μm.

**Additional file 3.** Proliferation and differentiation of satellite cells from eight different muscle groups from four month- or two-year-old Pax7-ZsGreen mice. (a) Cloning efficiency – the number of wells of a 96-well plate containing cells after 8 days of culture under low oxygen conditions. (b) Proliferation and survival –the average number of nuclei in a colony. (c) Spontaneous differentiation – the percentage of nuclei in Myosin Heavy Chain positive cytoplasm. Shown are mean ± SE (n=6, except diaphragm where n=4). Results of p-value comparisons (two-tailed t tests) are indicated above each graph.

**Additional file 4.** Satellite cells identified by surface marker staining analyzed for ZsGreen expression. Representative FACS profile of the percentage of lineage negative, indicated marker double positive cells for the three most-commonly used antibodies to identify satellite cells that are ZsGreen+ (n=3).

**Additional file 5.** No change in surface marker staining of satellite cells in two-year-old Pax7-ZsGreen mice. (a) Representative FACS profile of the percentage of ZsGreen+ cells that are lineage negative, indicated marker double positive cells for the three most-commonly used antibodies to identify satellite cells (n=3). (b) Representative FACS profile of the percentage of lineage negative, double positive cells for the three most-commonly used antibodies to identify satellite cells that are ZsGreen+ (n=3).

**Additional file 6.** Analysis of extent of injury from15 μL of cardiotoxin injection. (a) H+E stained sections of TA muscle from 3 month old male mice, uninjured (left) or injured with 15 μL cardiotoxin (right), two weeks post-injury. The area of centronucleation, representing the injured and regenerated domain, is outlined. (b) Mean regenerated area after injury 15 μL cardiotoxin.

**Additional file 7.** No difference observed after transplant of satellite cells isolated from the diaphragm of four month- or two-year-old Pax7-ZsGreen mice. (a) Number of ZsGreen+ cells quantified by FACS six weeks after transplant of 900 ZsGreen+ cells from the diaphragm (n=5). (b) Number of Dystrophin+ fibers six weeks after transplant of 900 ZsGreen+ from the diaphragm (n=5). (c) Number of ZsGreen+ cells quantified by FACS fifteen weeks after transplant of 900 ZsGreen+ cells from the diaphragm (n=5). (d) Number of Dystrophin+ fibers fifteen weeks after transplant of 900 ZsGreen+ cells from the diaphragm (n=5). Data shown are mean ± SE. Statistical comparisons were performed using two-tailed t tests.

## Data Availability

The datasets used and/or analyzed during the current study are available from the corresponding author on reasonable request.

## Abbreviations

BSA: Bovine serum albumin
cGy: Centigray
CSA: Cross-sectional area
DAPI: 4,6-Diamidino-2-phenylindole
DMEM: Dulbecco’s modified Eagle’s medium
EDL: Extensor digitorum longus
FACS: Fluorescence-activated Cell Sorting
MHC: Myosin heavy chain
PBS: Phosphate-buffered saline
TA: Tibialis anterior
