# Supplementary material for: Preservation of satellite cell number and regenerative potential with age reveals locomotory muscle bias
Source: Skelet Muscle. 2021 Sep 4;11:22. doi: 10.1186/s13395-021-00277-2 (PMC8418011; doi:10.1186/s13395-021-00277-2)
Supplement: Supplementary file 1 — Additional file 1. FACS plots for the quantification of the total number of satellite cells in different muscle groups from four-month- and two-year-old Pax7-ZsGreen mice. Representative FACS profiles for the gating for ZsGreen+ cells from eight different muscle groups – TA, EDL, Soleus, Gastrocnemius, Diaphragm, Psoas, Triceps and Masseter (n=6, except diaphragm where n=4). [file 13395_2021_277_MOESM1_ESM.pdf]

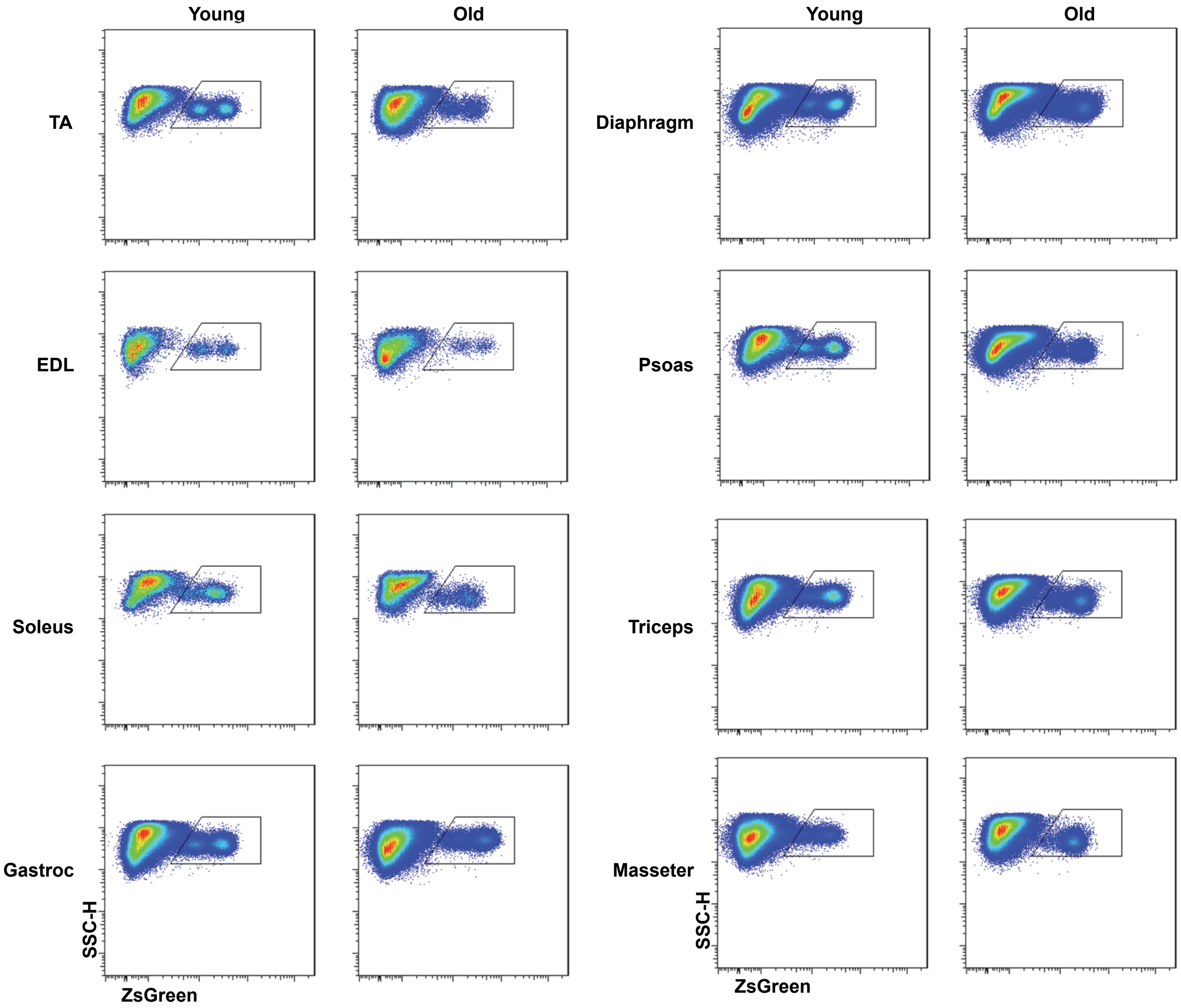

Arpke et al., Additional file 1

FACS plots for the quantification of the total number of satellite cells in different muscle groups from four-month- and two-year-old Pax7-ZsGreen mice. Representative FACS profiles for the gating for ZsGreen+ cells from eight different muscle groups – TA, EDL, Soleus, Gastrocnemius, Diaphragm, Psoas, Triceps and Masseter (n=6, except diaphragm where n=4).
