# Supplementary material for: Preservation of satellite cell number and regenerative potential with age reveals locomotory muscle bias
Source: Skelet Muscle. 2021 Sep 4;11:22. doi: 10.1186/s13395-021-00277-2 (PMC8418011; doi:10.1186/s13395-021-00277-2)
Supplement: Supplementary file 2 — Additional file 2. Representative immunostaining of Pax7+ cells, together with laminin and DAPI, in young (3 month) vs. old (22 months) TA muscle sections (above), and young vs. old masseter muscle sections (below). Scale bar: 50 μm. [file 13395_2021_277_MOESM2_ESM.pdf]

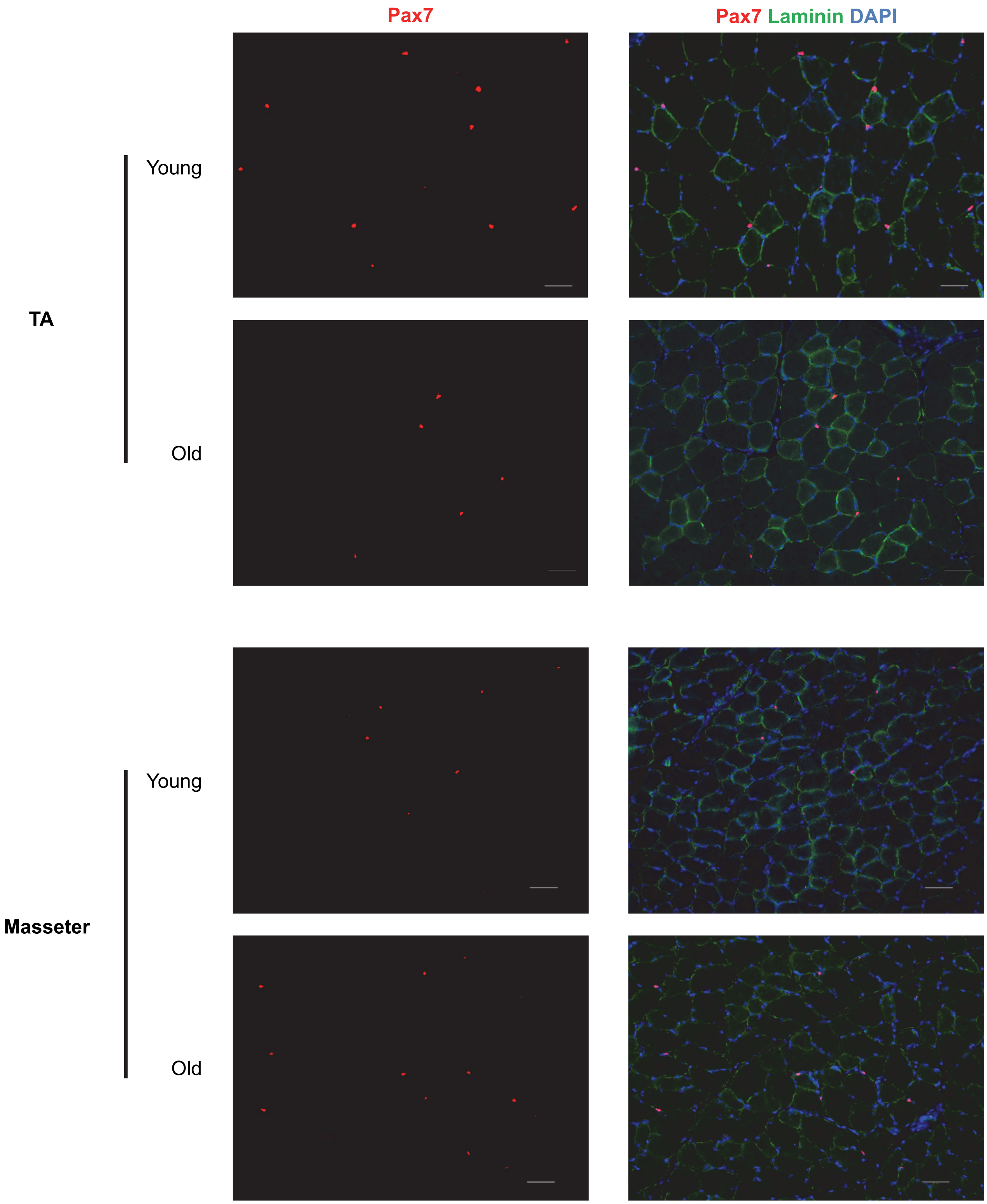

Arpke et al., Additional file 2

Representative immunostaining of Pax7+ cells, together with laminin and DAPI, in young (3 month) vs. old (22 months) TA muscle sections (above), and young vs. old masseter muscle sections (below). Scale bar: 50 µm.
