# Supplementary material for: Preservation of satellite cell number and regenerative potential with age reveals locomotory muscle bias
Source: Skelet Muscle. 2021 Sep 4;11:22. doi: 10.1186/s13395-021-00277-2 (PMC8418011; doi:10.1186/s13395-021-00277-2)
Supplement: Supplementary file 3 — Additional file 3. Proliferation and differentiation of satellite cells from eight different muscle groups from four month- or two-year-old Pax7-ZsGreen mice. (a) Cloning efficiency – the number of wells of a 96-well plate containing cells after 8 days of culture under low oxygen conditions. (b) Proliferation and survival –the average number of nuclei in a colony. (c) Spontaneous differentiation – the percentage of nuclei in Myosin Heavy Chain positive cytoplasm. Shown are mean ± SE (n=6, except diaphragm where n=4). Results of p-value comparisons (two-tailed t tests) are indicated above each graph. [file 13395_2021_277_MOESM3_ESM.pdf]

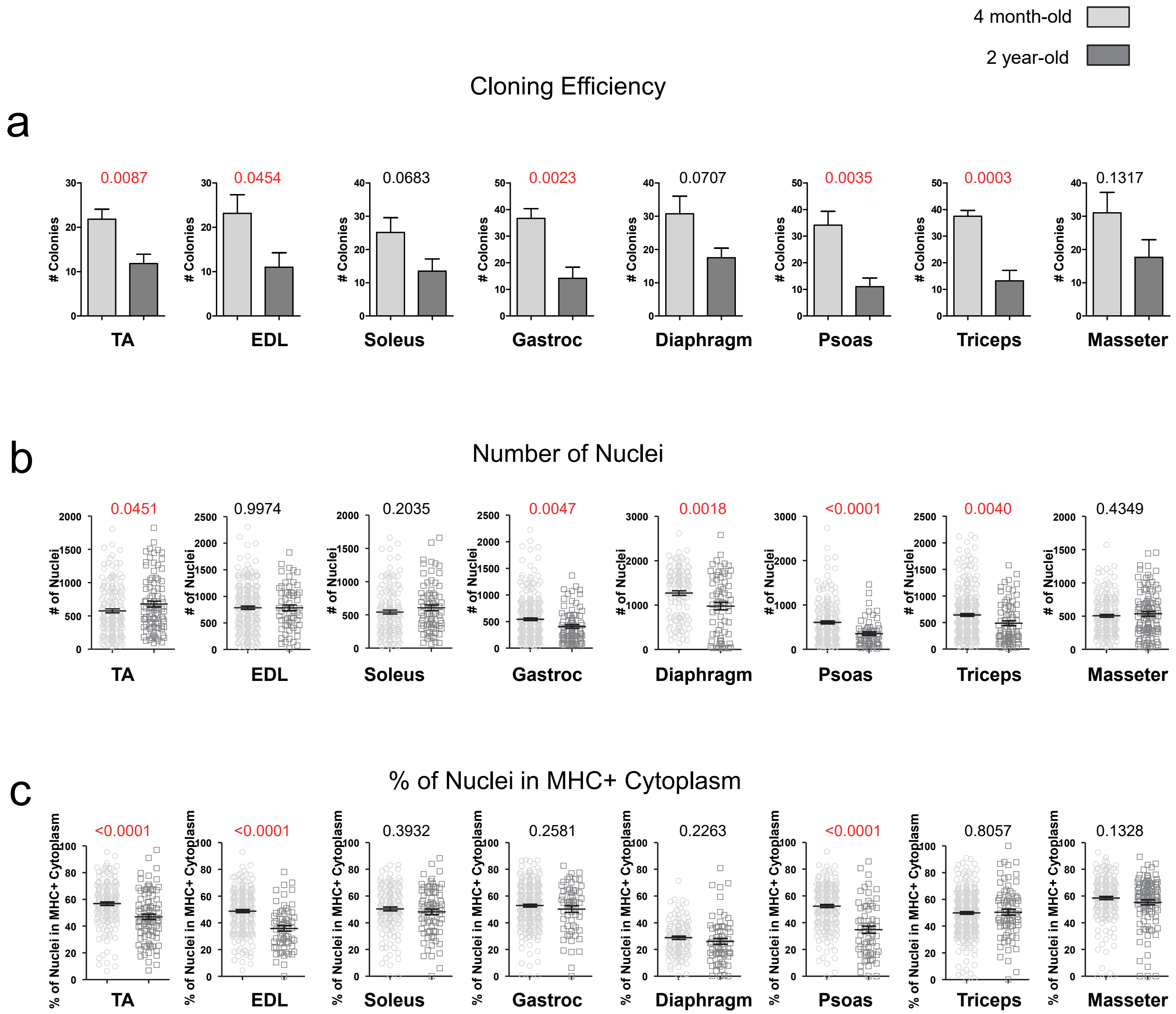

Arpke et al., Additional file 3

Proliferation and differentiation of satellite cells from eight different muscle groups from four-month- or two-year-old Pax7-ZsGreen mice. (a) Cloning efficiency – the number of wells of a 96-well plate containing cells after 8 days of culture under low oxygen conditions. **(b)** Proliferation and survival – the average number of nuclei in a colony. **(c)** Spontaneous differentiation – the percentage of nuclei in Myosin Heavy Chain positive cytoplasm. Shown are mean ± SE (n=6, except diaphragm where n=4). Results of p-value comparisons (two-tailed t tests) are indicated above each graph.
