# Supplementary material for: Preservation of satellite cell number and regenerative potential with age reveals locomotory muscle bias
Source: Skelet Muscle. 2021 Sep 4;11:22. doi: 10.1186/s13395-021-00277-2 (PMC8418011; doi:10.1186/s13395-021-00277-2)
Supplement: Supplementary file 4 — Additional file 4. Satellite cells identified by surface marker staining analyzed for ZsGreen expression. Representative FACS profile of the percentage of lineage negative, indicated marker double positive cells for the three most-commonly used antibodies to identify satellite cells that are ZsGreen+ (n=3). [file 13395_2021_277_MOESM4_ESM.pdf]

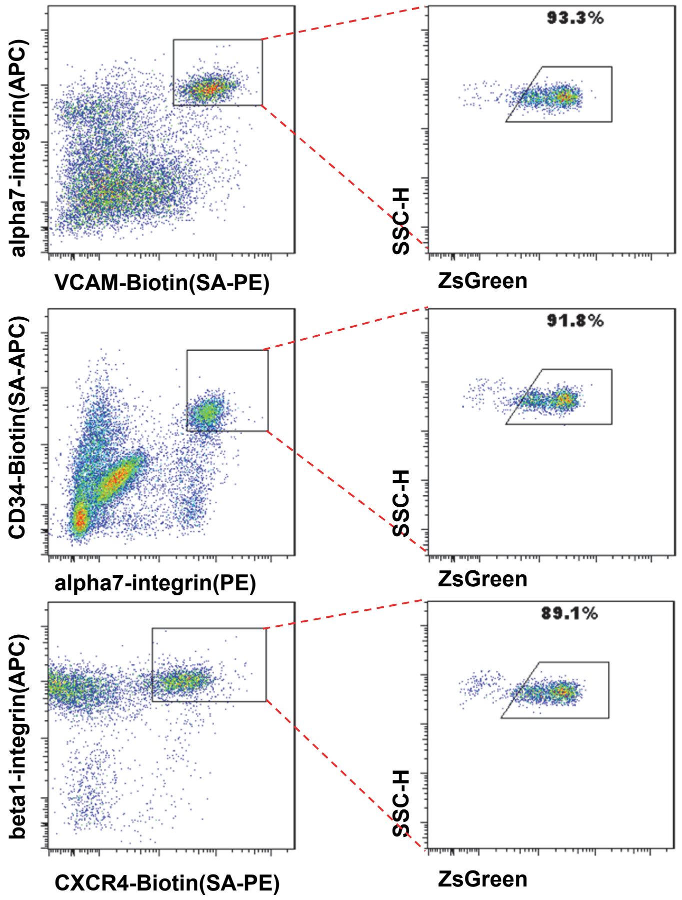

Arpke et al., Additional file 4

Satellite cells identified by surface marker staining analyzed for ZsGreen expression.
Representative FACS profile of the percentage of lineage negative, indicated marker double positive cells for the three most-commonly used antibodies to identify satellite cells that are ZsGreen+ (n=3).
