# Supplementary material for: Preservation of satellite cell number and regenerative potential with age reveals locomotory muscle bias
Source: Skelet Muscle. 2021 Sep 4;11:22. doi: 10.1186/s13395-021-00277-2 (PMC8418011; doi:10.1186/s13395-021-00277-2)
Supplement: Supplementary file 5 — Additional file 5. No change in surface marker staining of satellite cells in two-year-old Pax7-ZsGreen mice. (a) Representative FACS profile of the percentage of ZsGreen+ cells that are lineage negative, indicated marker double positive cells for the three most-commonly used antibodies to identify satellite cells (n=3). (b) Representative FACS profile of the percentage of lineage negative, double positive cells for the three most-commonly used antibodies to identify satellite cells that are ZsGreen+ (n=3). [file 13395_2021_277_MOESM5_ESM.pdf]

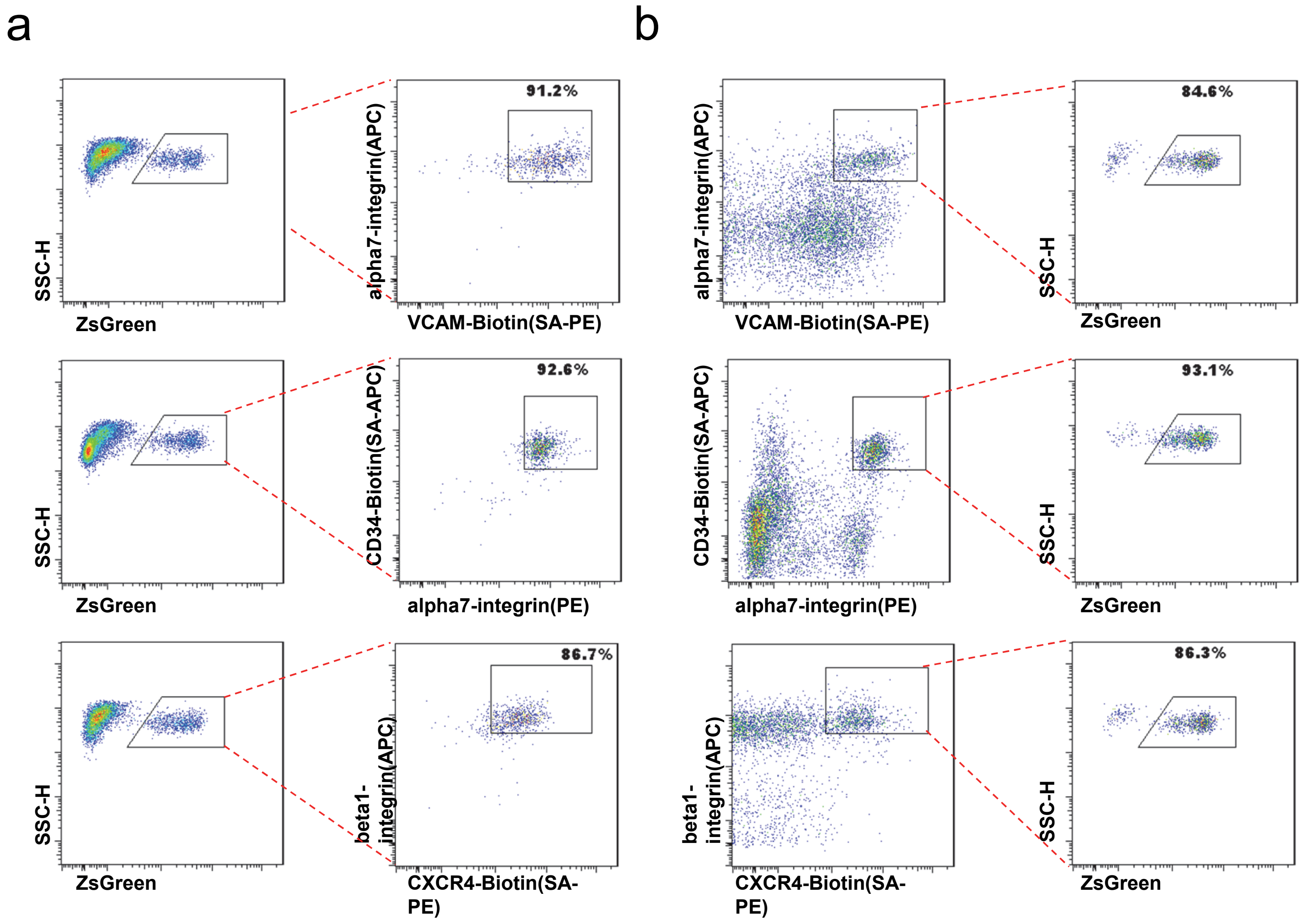

Arpke et al., Additional file 5

No change in surface marker staining of satellite cells in two-year-old Pax7-ZsGreen mice. (a) Representative FACS profile of the percentage of ZsGreen+ cells that are lineage negative, indicated marker double positive cells for the three most-commonly used antibodies to identify satellite cells (n=3). **(b)** Representative FACS profile of the percentage of lineage negative, double positive cells for the three most-commonly used antibodies to identify satellite cells that are ZsGreen+ (n=3).
