# Supplementary material for: Preservation of satellite cell number and regenerative potential with age reveals locomotory muscle bias
Source: Skelet Muscle. 2021 Sep 4;11:22. doi: 10.1186/s13395-021-00277-2 (PMC8418011; doi:10.1186/s13395-021-00277-2)
Supplement: Supplementary file 6 — Additional file 6. Analysis of extent of injury from15 μL of cardiotoxin injection. (a) H+E stained sections of TA muscle from 3 month old male mice, uninjured (left) or injured with 15 μL cardiotoxin (right), two weeks post-injury. The area of centronucleation, representing the injured and regenerated domain, is outlined. (b) Mean regenerated area after injury 15 μL cardiotoxin. [file 13395_2021_277_MOESM6_ESM.pdf]

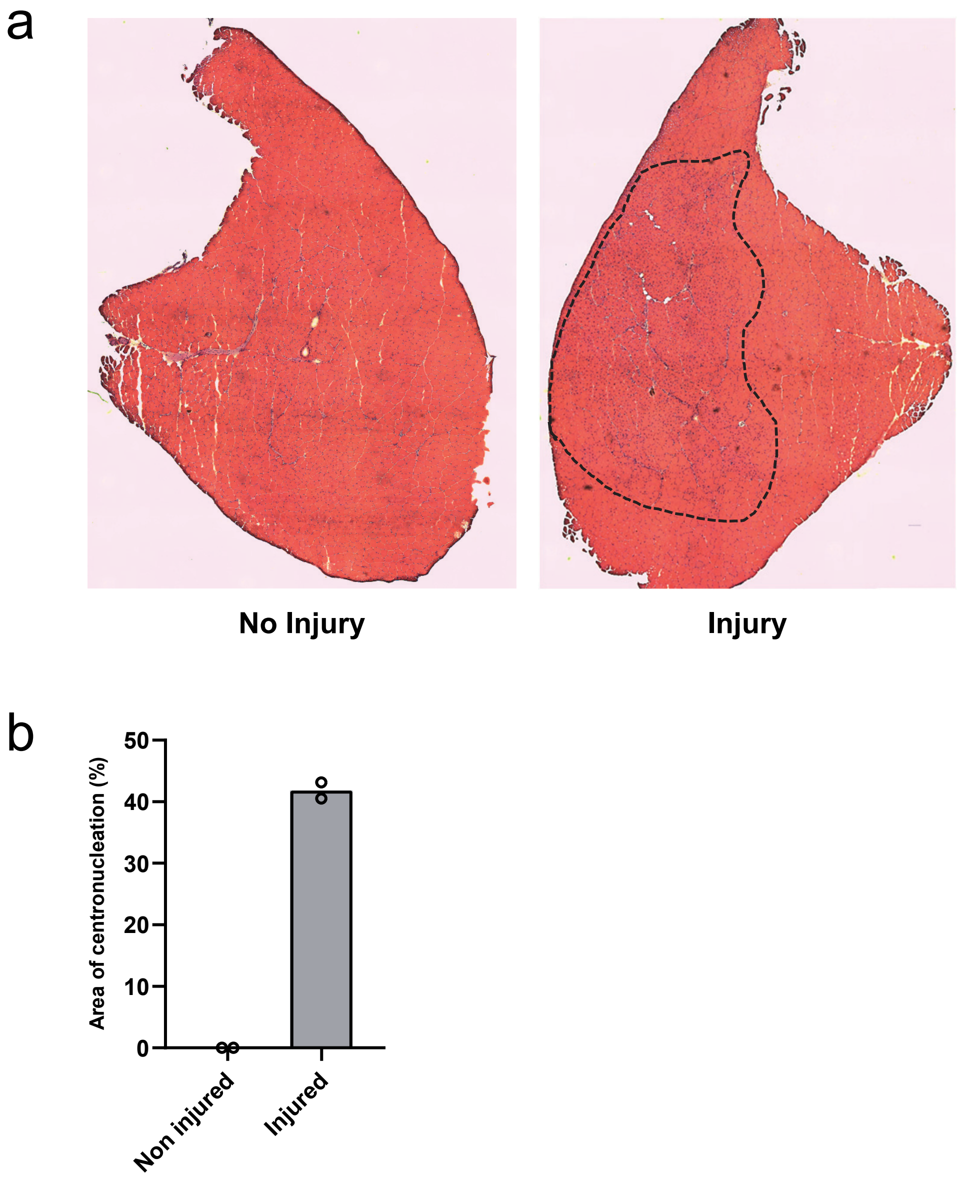

Arpke et al., Additional file 6

Analysis of extent of injury from15 μL of cardiotoxin injection. (a) H+E stained sections of TA muscle from 3 month old male mice, uninjured (left) or injured with 15 μL cardiotoxin (right), two weeks post-injury. The area of centronucleation, representing the injured and regenerated domain, is outlined. (**b)** Mean regenerated area after injury 15 μL cardiotoxin.
