# Supplementary material for: Preservation of satellite cell number and regenerative potential with age reveals locomotory muscle bias
Source: Skelet Muscle. 2021 Sep 4;11:22. doi: 10.1186/s13395-021-00277-2 (PMC8418011; doi:10.1186/s13395-021-00277-2)
Supplement: Supplementary file 7 — Additional file 7. No difference observed after transplant of satellite cells isolated from the diaphragm of four month- or two-year-old Pax7-ZsGreen mice. (a) Number of ZsGreen+ cells quantified by FACS six weeks after transplant of 900 ZsGreen+ cells from the diaphragm (n=5). (b) Number of Dystrophin+ fibers six weeks after transplant of 900 ZsGreen+ from the diaphragm (n=5). (c) Number of ZsGreen+ cells quantified by FACS fifteen weeks after transplant of 900 ZsGreen+ cells from the diaphragm (n=5). (d) Number of Dystrophin+ fibers fifteen weeks after transplant of 900 ZsGreen+ cells from the diaphragm (n=5). Data shown are mean ± SE. Statistical comparisons were performed using two-tailed t tests. [file 13395_2021_277_MOESM7_ESM.pdf]

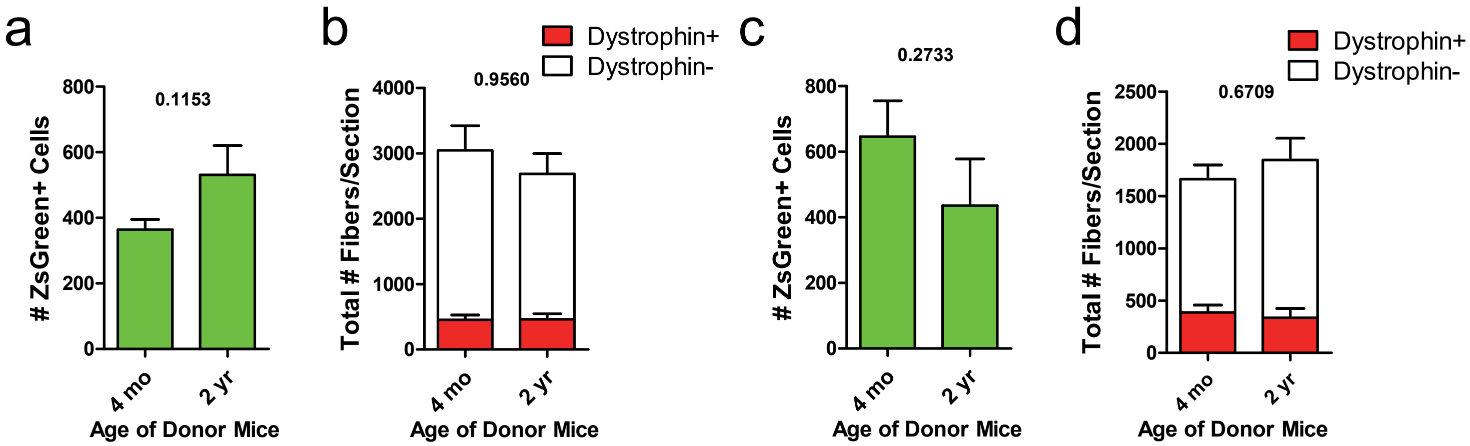

Arpke et al., Additional file 7

No difference observed after transplant of satellite cells isolated from the diaphragm of four-month- or two-year-old Pax7-ZsGreen mice. (a) Number of ZsGreen+ cells quantified by FACS six weeks after transplant of 900 ZsGreen+ cells from the diaphragm (n=5). **(b)** Number of Dystrophin+ fibers six weeks after transplant of 900 ZsGreen+ from the diaphragm (n=5). **(c)** Number of ZsGreen+ cells quantified by FACS fifteen weeks after transplant of 900 ZsGreen+ cells from the diaphragm (n=5). **(d)** Number of Dystrophin+ fibers fifteen weeks after transplant of 900 ZsGreen+ cells from the diaphragm (n=5). Data shown are mean ± SE. Statistical comparisons were performed using two-tailed t tests.
